# Speech Recognition Technology and Documentation Efficiency

**DOI:** 10.1001/jamanetworkopen.2025.1526

**Published:** 2025-03-24

**Authors:** Abdul R. Shour, Ronald Anguzu, Adedayo A. Onitilo

**Affiliations:** 1 Cancer Care and Research Center, Marshfield Clinic Research Institute, Marshfield Clinic Health System, Marshfield, Wisconsin; 2 Department of Pediatrics, University of Wisconsin–Madison School of Medicine and Public Health; 3 Now with Essentia Institute of Rural Health, Essentia, Duluth, Minnesota; 4 Institute for Health Equity, Medical College of Wisconsin, Milwaukee; 5 Oncology Service Line, Marshfield Clinic, Weston, Wisconsin; 6 Department of Medicine, University of Wisconsin–Madison School of Medicine and Public Health

## Abstract

This cross-sectional study evaluates whether the use of a speech recognition tool is associated with documentation efficiency among clinicians at a rural US health care system.

## Introduction

Clinical documentation is crucial in ensuring high-quality health care delivery.^1^ Speech recognition (SR) technology, such as Dragon Medical One, emerged as a tool to streamline documentation processes, alleviate clinician workload, and address inefficiencies associated with manual transcription.^2^ Due to personnel and resource constraints, SR technology adoption is essential for workflow efficiency in rural settings, such as the Marshfield Clinic Health System (MCHS). Despite the growing adoption of SR, its impact remains controversial, with variations in error rates and satisfaction.^2,3,4,5^ There is a need for in-depth research on how SR tools affect documentation efficiency across various medical specialties and clinician demographics. We analyzed the association between SR tool usage and documentation efficiency using objective efficiency metrics (lines documented per hour) across various medical specialties, addressing limitations of prior studies by focusing on a rural context and providing novel insights into SR’s applicability beyond urban and specialty-specific settings.

## Methods

This cross-sectional study was conducted following the STROBE guidelines^6^ within MCHS, covering Wisconsin and Michigan’s Upper Peninsula, from September 1, 2018, to December 31, 2020. The MCHS institutional review board deemed this study exempt from review and the requirement for informed consent due to its retrospective nature and use of deidentified data. Of 1373 clinicians, 1124 were included after excluding missing data.

The outcome was documentation efficiency, measured as lines documented per hour. The dependent variable was the percentage of SR tool usage. Covariates included age, sex, professional degree, years in practice, credentials, and departments: surgical specialties (cardiovascular surgery, neurosurgery, orthopedics, urology), medical specialties (cardiology, dermatology, oncology), pediatrics, behavioral health, obstetrics and gynecology, and other departments (emergency medicine, hospitalist, physical therapy, wound healing). These are consistent with MCHS’s classification system and the American Board of Medical Specialties’ criteria.

Descriptive statistics and bivariate and multivariable analyses using negative binomial regression were utilized adjusting for covariates. Interaction terms tested differences across departments but were not statistically significant. Analyses used Stata version 18.5 (StataCorp) with 2-sided *P* ≤ .05 considered significant; analyses were conducted between January 2021 and September 2024.

## Results

Among 1124 clinicians (Table 1), clinicians had a mean (SD) age of 47.68 (12.21) years, with 52.0% female and 48.0% male. The mean (SD) number of lines documented per hour was 428.35 (188.12), and the mean (SD) percentage of SR tool usage was 34.55% (46.79%).

**Table 1.** Demographic and Professional Characteristics of 1124 Clinicians

Characteristic	Clinicians, No. (%) (N = 1124)^a^
Lines per hour, mean (SD) [range]	428.35 (188.12) [0-1878.73]
Skewness	0.748
Kurtosis	8.102
SR tool usage, mean (SD) [range], %	34.55 (46.79) [0-100]
Skewness	0.653
Kurtosis	1.450
Clinician’s age, mean (SD) [range], y	47.68 (12.21) [22-83]
Skewness	0.107
Kurtosis	2.036
Age categories	
<29 y	58 (7.1)
30-39 y	37 (4.5)
40-49 y	235 (28.7)
50-59 y	275 (33.6)
≥60 y	214 (26.1)
Sex	
Female	584 (52.0)
Male	540 (48.0)
Professional degree	
Internationally trained MD	237 (22.1)
US-trained MD	834 (77.9)
Clinical practice years, mean (SD) [range]	10.71 (9.52) [0-46]
Skewness	0.941
Kurtosis	3.028
Years in practice categories	
≤10 y	666 (59.4)
11-20 y	264 (23.6)
≥21 y	191 (17.0)
Credential	
Nonphysician or allied health (NP, PA, other)	472 (42.1)
MD	649 (57.9)
Medical department	
Primary care (internal medicine and family medicine)	166 (14.8)
Surgical specialties	169 (15.0)
Medical specialties	345 (30.7)
Pediatrics	112 (10.0)
Behavioral health	58 (5.2)
Obstetrics and gynecology	49 (4.4)
Other	225 (20.0)

Abbreviations: MD, medical doctor; NP, nurse practitioner; PA, physician assistant; SR, speech recognition.

^a^ Totals may not sum to 1124 due to missing data in some categories. Percentages are calculated based on the number of clinicians for whom data were available. Quality of evidence was rated as 4.

In the bivariate analysis (Table 2), SR tool usage was significantly associated with higher documentation efficiency (coefficient, 0.003; 95% CI, 0.002 to 0.004; *P* < .001). In the multivariable analysis, SR tool usage remained significantly associated with higher documentation efficiency (coefficient, 0.003; 95% CI, 0.002 to 0.003; *P* < .001). Clinicians in surgical specialties (coefficient, 0.153; 95% CI, 0.059 to 0.247; *P* = .001) and other departments (coefficient, 0.125; 95% CI, 0.040 to 0.211; *P* = .004) documented more lines per hour than primary care (internal medicine and family medicine). Age remained negatively associated with documentation efficiency (coefficient, –0.007; 95%CI, –0.009 to –0.004; *P* < .001). Male clinicians documented more lines per hour than female clinicians (coefficient, 0.111; 95% CI, 0.056 to 0.166; *P* < .001).

**Table 2.** Bivariate and Multivariable Analyses of Factors Associated with Documentation Efficiency^a^

Characteristic	Bivariate analysis		Multivariable analysis	
	Coefficient (95% CI)	*P* value	Coefficient (95% CI)	*P* value
SR tool usage, per 1% increase	0.003 (0.002 to 0.004)	<.001	0.003 (0.002 to 0.003)	<.001
Department category				
Primary care	[Reference]	NA	[Reference]	NA
Surgical specialties	0.150 (0.031 to 0.270)	.01	0.153 (0.059 to 0.247)	.001
Medical specialties	−0.010 (−0.113 to 0.093)	.85	−0.020 (−0.101 to 0.062)	.64
Pediatrics	0.016 (−0.118 to 0.149)	.82	−0.013 (−0.128 to 0.102)	.82
Behavioral health	−0.041 (−0.207 to 0.126)	.63	0.012 (−0.108 to 0.133)	.84
Obstetrics and gynecology	0.066 (−0.112 to 0.243)	.47	0.041 (−0.072 to 0.153)	.48
Other	0.215 (0.104 to 0.327)	<.001	0.125 (0.040 to 0.211)	.004
Clinician’s age, y	−0.009 (−0.012 to −0.007)	<.001	−0.007 (−0.009 to −0.004)	<.001
Sex				
Female	[Reference]	NA	[Reference]	NA
Male	0.074 (0.008 to 0.140)	.03	0.111 (0.056 to 0.166)	<.001
Professional degree				
Internationally trained MD	[Reference]	NA	[Reference]	NA
US-trained MD	0.043 (−0.041 to 0.126)	.32	0.068 (−0.002 to 0.137)	.06
Years in clinical practice	−0.009 (−0.012 to −0.007)	<.001	−0.006 (−0.009 to −0.002)	.002
Credential				
Nonphysician/allied health	[Reference]	NA	[Reference]	NA
MD	−0.004 (−0.071 to 0.063)	.90	0.032 (−0.030 to 0.093)	.31

Abbreviations: MD, medical doctor; NA, not applicable; SR, speech recognition.

^a^ Quality of evidence was rated as 4.

## Discussion

In this study, higher SR tool usage was associated with a 0.25% increase in lines documented per hour for each 1% increase in usage. Clinicians in surgical specialties and other departments documented significantly more lines per hour than those in primary care. Documentation efficiency was higher among younger and male clinicians.

One limitation is the potential for selection bias, as the study was conducted in a single rural health care system, which may limit the generalizability of results to urban or more diverse health care settings.^3^ However, MCHS includes 11 hospitals and 60 clinics. Additionally, documentation efficiency as lines per hour may not capture time savings or workflow improvements. This study did not account for document types, which may affect efficiency; future research should explore this to better understand SR tool utilization. Unmeasured factors, such as workload or SR familiarity, may affect results.^2^ The cross-sectional design precludes causal inference, but these findings align with previous literature on SR technology, extending the focus to clinician productivity over error rates.^4,5^ Further research is needed to validate these findings in different clinical environments.

## Notes

Supplement.
Data Sharing Statement
