# Supplementary material for: Speech Recognition Technology and Documentation Efficiency
Source: JAMA Netw Open. 2025 Mar 24;8(3):e251526. doi: 10.1001/jamanetworkopen.2025.1526 (PMC11934006; doi:10.1001/jamanetworkopen.2025.1526)
Supplement: Supplement. — Data Sharing Statement [file jamanetwopen-e251526-s001.pdf]

Data Sharing Statement

Shour. Speech Recognition Technology and Documentation Efficiency. *JAMA Netw Open*. Published March 24, 2025. doi:10.1001/jamanetworkopen.2025.1526

Data
Data available: No

Additional Information
Explanation for why data not available: Data sharing is not applicable to this article as no datasets were generated or analyzed during the current study beyond those reported in the article.
